# In Vivo Efficacy of Rezafungin, Anidulafungin, Caspofungin, and Micafungin against Four *Candida auris* Clades in a Neutropenic Mouse Bloodstream Infection Model

**DOI:** 10.3390/jof10090617

**Published:** 2024-08-29

**Authors:** Dávid Balázsi, Zoltán Tóth, Jeffrey B. Locke, Andrew M. Borman, Lajos Forgács, Noémi Balla, Fruzsina Kovács, Renátó Kovács, Chiaki Amano, Tugba Ilay Baran, László Majoros

**Affiliations:** 1 Medical Microbiology, Clinical Center, Faculty of Medicine, University of Debrecen, 4032 Debrecen, Hungary; 2 Doctoral School of Pharmaceutical Sciences, University of Debrecen, 4032 Debrecen, Hungary; 3 Cidara Therapeutics, Inc., 6310 Nancy Ridge Dr., Suite 101, San Diego, CA 92121, USA; 4 UK National Mycology Reference Laboratory, UK Health Security Agency, Science Quarter, Southmead Hospital, Bristol BS10 5NB, UK; 5 Medical Research Council Centre for Medical Mycology (MRC CMM), University of Exeter, Exeter EX4 4QD, UK; 6 Department of Medical Microbiology, Faculty of Medicine, University of Debrecen, 4032 Debrecen, Hungary

**Keywords:** echinocandins, *Candida auris*, murine model

## Abstract

Objectives: Rezafungin is the first new drug approved to treat candidaemia and invasive candidiasis in more than 10 years. However, data are scant on the in vivo efficacy of rezafungin and the other three approved echinocandins against different *Candida auris* clades. Methods: This study involved 10 isolates representing 4 *C. auris* clades: South Asian (n = 2), East Asian (n = 2), South African (n = 2), and South American (n = 4, including 2 environmental isolates). In the lethality experiment and fungal tissue burden experiment (kidney, heart, and brain), cyclophosphamide-treated BALB/c male mice were intravenously infected (10^7^ and 8 × 10^6^ colony-forming units [CFU]/mouse, respectively). A 20 mg/kg dose of rezafungin was administered on days 1, 3, and 6. Alternatively, beginning 24 h post-infection, mice received 3 mg/kg of caspofungin, 5 mg/kg of micafungin, or 5 mg/kg of anidulafungin once daily for 6 days. Results: Regardless of isolate and clade, all echinocandin regimens improved survival after 21 days (*p* = 0.0041 to *p* < 0.0001). All echinocandins frequently produced >3-log mean CFU/g decreases in the fungal kidney and heart burdens, although some of these decreases were not statistically significant. Rezafungin, regardless of clade, produced 3–5 and 2–4 log CFU/g decreases in the kidney and heart burdens, respectively. Echinocandins did not inhibit fungal growth in the brain. Histopathological examination performed on day 7 showed no fungal cells in the heart and kidneys of rezafungin-treated mice and to a lesser extent, caspofungin-treated mice, regardless of the clinical isolate. All echinocandin-treated mice showed medium and/or large foci of fungal cells in their cerebrum or cerebellum. Conclusions: Regardless of the *C. auris* clade, rezafungin activity in vivo was comparable to or improved over that of the three previously approved echinocandins.

## 1. Introduction

*Candida (Candidiozyma*) *auris*, a globally distributed yeast, is classified as a critical priority fungal pathogen by the World Health Organization [1]. The first whole-genome sequencing data of global clinical isolates identified four distinct clades (South Asian, East Asian, South African, and South American) that are responsible for hospital outbreaks and invasive infections in critically ill patients. Most clinical isolates were resistant to fluconazole and, to a lesser extent, amphotericin B [2,3]. Echinocandins are recommended as first-line antifungal agents for the treatment of candidaemia and invasive candidiasis caused by different *Candida* species, including *C. auris* [4,5]. The most important drivers of echinocandin efficacy are the free-drug area under the concentration–time curve from 0 to 168 h (*f*AUC_0–168_) divided by the minimum inhibitory concentration (MIC) (i.e., the *f*AUC_0–168_/MIC ratio) and the free-drug maximum concentration (C_max_) divided by the MIC (i.e., the C_max_/MIC ratio) [6,7]. However, the standard dosing regimens of previously approved echinocandins do not always achieve the necessary therapeutic levels for *Candida* species with elevated MICs and for the invasive candidiasis of deep tissues and organs. This situation often leads to therapeutic failure in critically ill patients [8,9,10,11,12].

Rezafungin (Rezzayo™; Cidara Therapeutics, Inc., San Diego, CA, USA) is the first new drug approved to treat candidaemia and invasive candidiasis in more than 10 years [3,4,13]. Rezafungin is a once-weekly, next-generation echinocandin with excellent in vitro and in vivo activity against the clinically important *Candida* species. Rezafungin has been approved for the treatment of candidaemia and invasive candidiasis in patients older than 18 years who have limited or no alternative treatment options [13]. However, there is little information on the in vivo efficacy of the four approved echinocandins against the four principal *C. auris* clades.

This study was performed to compare the in vivo efficacy of rezafungin, anidulafungin, caspofungin, and micafungin against *C. auris* isolates belonging to South Asian, East Asian, South African, and South American lineages in a neutropaenic mouse model.

## 2. Materials and Methods

### 2.1. Isolates

This study involved 10 isolates representing 4 *C. auris* clades derived from our previous studies: South Asian (n = 2), East Asian (n = 2), South African (n = 2), and South American (n = 4, including 2 environmental isolates). These isolates are listed in Table 1 [14,15,16,17]. The *C. auris* isolates were identified by a combination of ribosomal DNA gene sequencing targeting the 28S rRNA and/or ITS1 regions; this process was also used for clade delineation [18,19,20]. Based on previous whole-genome sequencing data, all isolates were *FKS* wild-type [17]. Four of the ten isolates produced aggregates in saline (Table 1) [16]. Two days before the experiments, the isolates were sub-cultured using Sabouraud agar and screened on CHROMagar™ Candida (CHROMagar, Paris, France) to ensure the purity of *Candida* isolates [14,15,16,17]. Echinocandin MIC values were determined using the broth microdilution method according to the CLSI M27-Ed4 guidelines in RPMI-1640 medium [21,22]. The MICs of anidulafungin, caspofungin, and micafungin were not higher than the suggested CDC tentative breakpoints for *C. auris* (Table 1) [5]. The MIC of rezafungin was lower than the CLSI susceptible breakpoint [22].

### 2.2. Lethality Experiments

BALB/c male mice (23–25 g) were administered intraperitoneal cyclophosphamide (Endoxan; Baxter Hungary Kft., Budapest, Hungary) 4 days before infection (150 mg/kg) and 1 day before infection (100 mg/kg). Immunosuppression was continued by administration of 100 mg/kg of cyclophosphamide every third day until the end of the experiment on day 21 [16,23]. The Guidelines for the Care and Use of Laboratory Animals were strictly followed during maintenance of the animals. All experiments were approved by the Animal Care Committee of the University of Debrecen (permission no. 12/2019).

The mice (groups of 10 mice/isolate) were intravenously infected through the lateral tail vein (day 0). The infectious dose was 10^7^ colony-forming units (CFU)/mouse, administered in volumes of 0.2 mL. Inoculum densities were confirmed by plating serial dilutions on Sabouraud agar [16,23].

Treatments with echinocandins began 24 h post-infection (day 1). A 20 mg/kg dose of rezafungin was administered on days 1, 3, and 6. Alternatively, beginning at 24 h post-infection, the mice received once-daily treatment for 6 days with 3 mg/kg of caspofungin (Cancidas^®^; Merck & Co., Inc., Kenilworth, NJ, USA), 5 mg/kg of micafungin (Mycamine^®^; Sandoz/Novartis, Basel, Switzerland), or 5 mg/kg of anidulafungin (Eraxis^®^; Pfizer, New York, NY, USA) [24,25,26]. All treatments were given intraperitoneally in a 0.5 mL volume. These doses mimic the human once-weekly dosing regimen for rezafungin and the daily dosing regimens for anidulafungin, caspofungin, and micafungin [4,6,13]. The control groups were administered saline. The mice were monitored for survival at least twice a day for 21 days. Animals that became immobile or showed signs of severe illness were euthanized and recorded as having died the following day. Survival rates were compared using the Kaplan–Meier log rank test. Statistical tests were performed using GraphPad Prism 10.2.3 software (GraphPad Software, La Jolla, CA, USA) [16,23].

### 2.3. Fungal Tissue Burden Experiments

At the beginning of therapy, the fungal burdens in the kidney, heart, and brain were determined after dissection of four untreated mice for each of the following isolates: 196 (South Asian clade), 12372 (East Asian clade), 2 (South African clade), I-156 (South American clade, from Israel), and 13112 (South American clade, from Colombia, hospital environment) (day 1 control burden) [23].

The experimental design was similar to that employed in the lethality experiments. Each group included seven mice. On day 7, five mice were sacrificed; both kidneys, the heart, and the brain were removed from each animal, weighed, and homogenized aseptically in 1 mL of saline; the resulting tissue suspension was serially diluted [23]. The fungal tissue burden was determined by quantitative culturing. The lower limit of detection was 100 CFU/g of tissue. The mean fungal tissue burdens produced by the same organs were compared using the Kruskal–Wallis test with Dunn’s post-test [23].

### 2.4. Histopathology

Two mice from both the treatment and control groups of the fungal tissue burden experiments were used for histopathological examination on day 7. Organs (heart, both kidneys, and brain) were fixed in formalin and embedded in paraffin. Tissue sections (4 µm) were stained with periodic acid–Schiff (PAS) reaction with haematoxylin nuclear staining [16,23].

## 3. Results

### 3.1. Lethality Experiments

**Figure 1:**
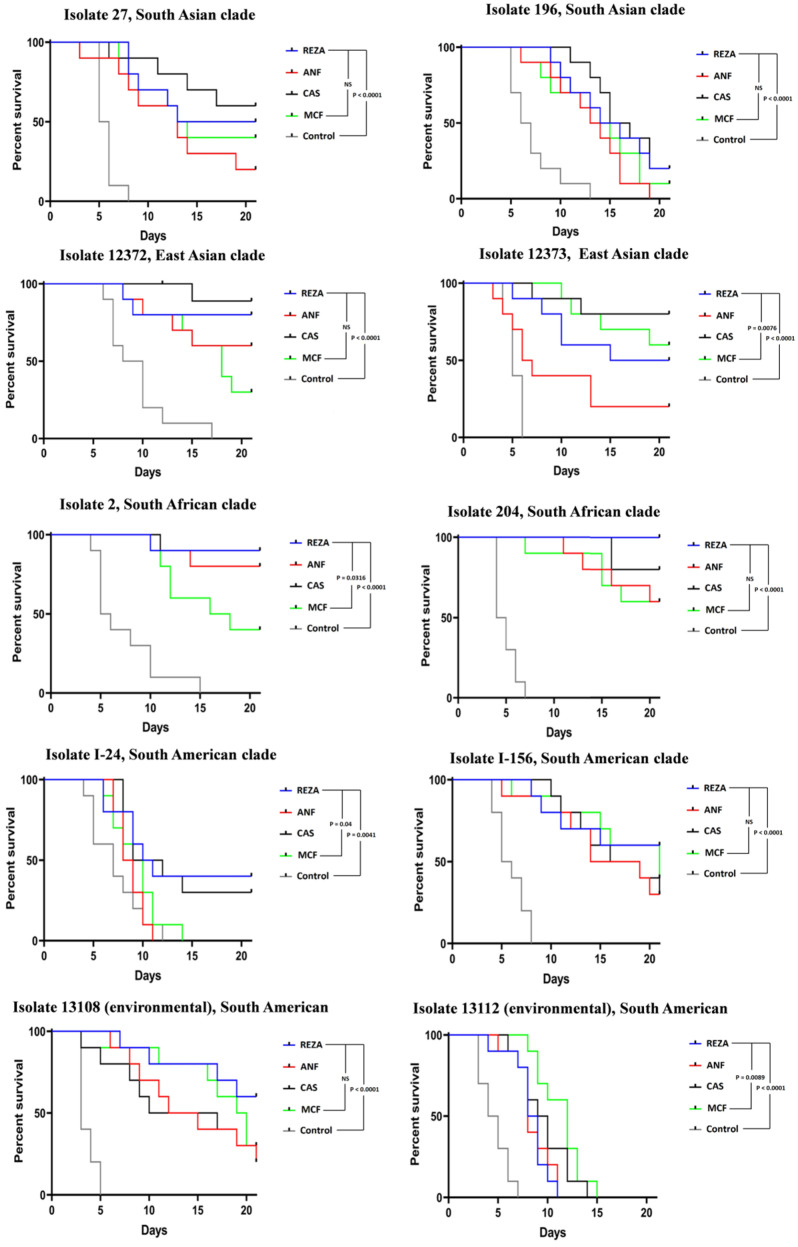
Survival of neutropaenic echinocandin-treated and control BALB/c mice infected with isolates from the four *Candida auris* clades: isolates 27 and 196 (South Asian clade), isolates CBS 12372 {12372} and CBS 12373 {12373} (East Asian clade), isolates 2 and 204 (South African clade), and isolates I-24 and I-156 (South American clade). Two environmental isolates from the South American clade were also tested: CDC B-13108 {13108} and CDC B-13112 {13112}. The infectious dose was 10^7^ CFU/mouse. A 20 mg/kg dose of rezafungin (REZA) was administered on days 1, 3, and 6. Additionally, beginning 24 h post-infection, the mice received 3 mg/kg of caspofungin (CAS) (Cancidas^®^), 5 mg/kg of micafungin (MCF) (Mycamine^®^), and 5 mg/kg of anidulafungin (ANF) (Eraxis^®^) once daily for 6 days. After 21 days, survival rates were compared using the Kaplan–Meier log rank test.

Regardless of the isolate and clade, all control mice died by day 17, and all echinocandin regimens improved the survival rate (*p* = 0.0041 to *p* < 0.0001) (Figure 1). Neither infected control mice nor echinocandin-treated mice showed signs of central nervous system involvement (i.e., ataxia) [23].

#### 3.1.1. South Asian Clade

All control mice infected with isolates 27 and 196 died by days 8 and 13, respectively. For the more virulent isolate 27, all mice died within 3 days (Figure 1). Echinocandins significantly increased the survival rate (*p* < 0.0001 for both isolates). On days 7 and 21, the survival rates of echinocandin-treated mice infected with the two isolates were 80–100% and 0–60%, respectively (Figure 1).

#### 3.1.2. East Asian Clade

All control mice inoculated with isolates 12372 and 12373 died by days 17 and 6, respectively. For isolate 12373, mortality occurred rapidly (100% mortality from days 4 to 6). All echinocandin regimens significantly increased the survival rates (*p* < 0.0001 for both isolates) (Figure 1). Caspofungin resulted in the highest survival rate (80%–90%) for mice infected with both isolates at day 21. The lowest survival rate occurred in mice treated with micafungin (30%, isolate 12372) and anidulafungin (20%, isolate 12373). In the case of isolate 12373, caspofungin-treated mice showed better survival than anidulafungin- and micafungin-treated mice and a significant difference was observed between therapeutic regimens (*p* = 0.0076).

#### 3.1.3. South African Clade

Isolate 204 was more virulent (100% mortality of control mice from days 4 to 7) than isolate 2 (gradual death of control mice from days 4 to 15). All four echinocandins were effective against both isolates (*p* < 0.0001 for both isolates) (Figure 1). Rezafungin-, caspofungin-, and anidulafungin-treated mice infected with isolate 2 exhibited 90%, 90%, and 80% survival at day 21, respectively. All of these survival rates were higher than that obtained with micafungin (40%) and a significant difference was observed between therapeutic regimens (*p* = 0.0316). Despite the rapid death of control mice infected with isolate 204, the four echinocandins resulted in 60–100% survival at day 21 (Figure 1).

#### 3.1.4. South American Clade

All four echinocandins improved the survival rates of mice with bloodstream isolates from Israel (*p* = 0.0041 and *p* < 0.0001 for isolates I-24 and I-156, respectively). The highest survival rate was associated with rezafungin (40% and 60% for mice infected with isolates I-24 and I-156, respectively), and all anidulafungin- and micafungin-treated mice died by days 11 and 14, respectively (Figure 1). Rezafungin- and caspofungin-treated mice infected with isolate I-24 showed higher survival than anidulafungin- and micafungin-treated mice (*p* = 0.04), and all anidulafungin-treated mice died before control mice (Figure 1). All control mice inoculated with the environmental isolates died by day 7, but the four echinocandins significantly improved the survival rates (*p* < 0.0001 for both isolates). Rezafungin and anidulafungin resulted in the highest (60%) and lowest (20%) survival rates, respectively, in mice infected with isolate 13108. Although, in the case of isolate 13112, caspofungin and micafungin resulted in higher survival rates than anidulafungin and rezafungin (*p* = 0.0089), all caspofungin- and micafungin-treated mice died by days 14 and 15, respectively (Figure 1). 

### 3.2. Fungal Burden Experiments

#### 3.2.1. Kidneys

**Figure 2:**
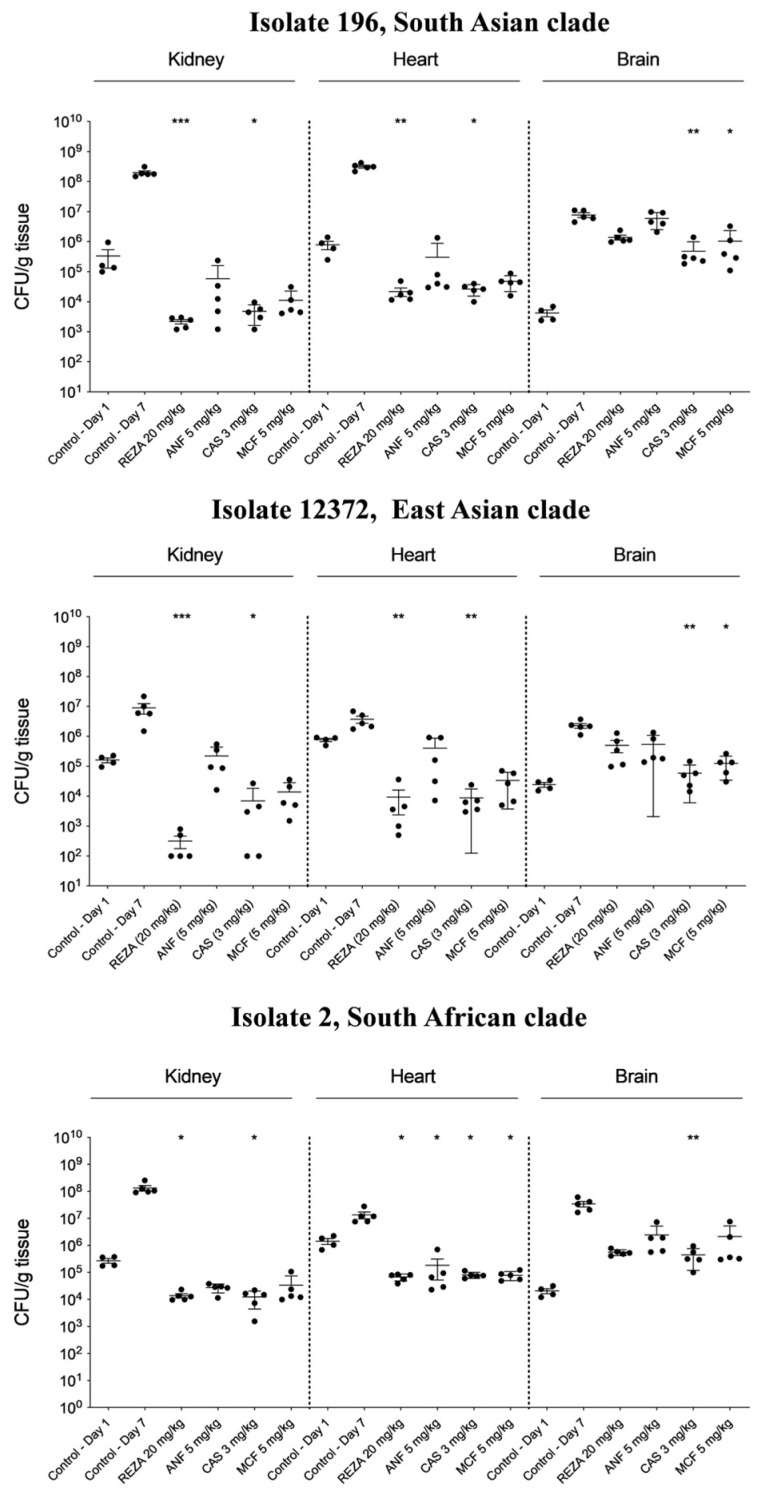

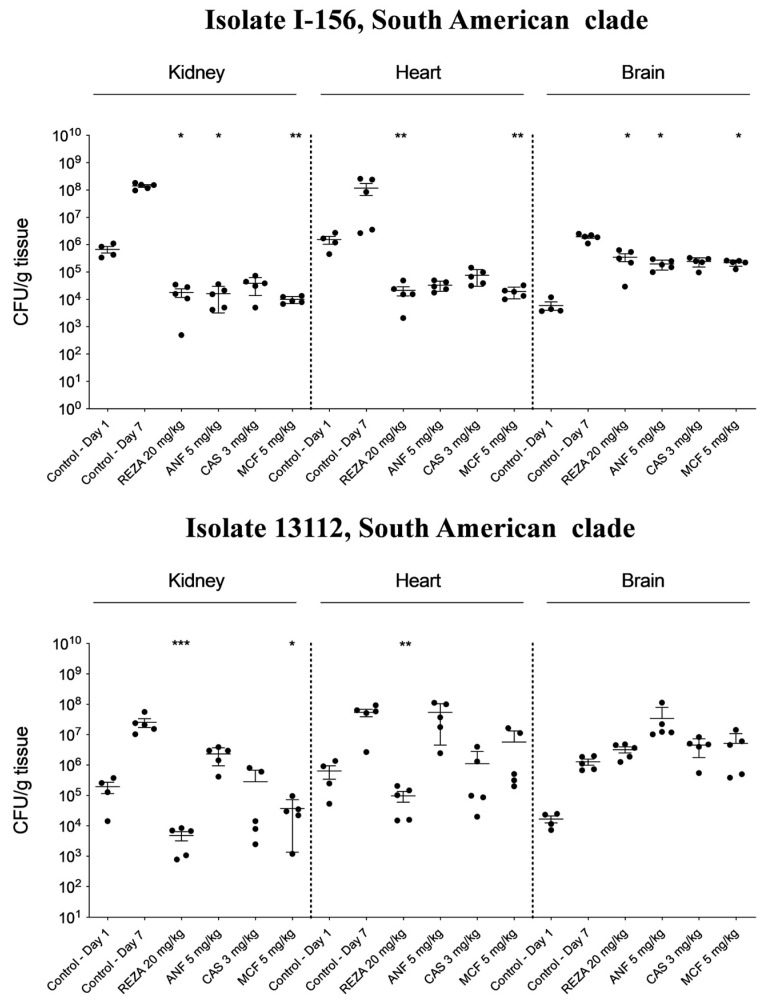
Fungal kidney, heart, and brain burdens were determined on days 1 and 7 with clinical isolates from the South Asian clade (isolate 196), East Asian clade (isolate CBS 12372), South African clade (isolate 2), and South American clade (isolate I-156). One environmental isolate from the South American clade was also tested (isolate CDC B-13112). The infectious dose was 8 × 10^6^ CFU/mouse. A 20 mg/kg dose of rezafungin (REZA) was administered on days 1, 3, and 6. Additionally, beginning 24 h post-infection, the mice received 3 mg/kg of caspofungin (CAS) (Cancidas^®^), 5 mg/kg of micafungin (MCF) (Mycamine^®^), and 5 mg/kg of anidulafungin (ANF) (Eraxis^®^) once daily for 6 days. The bars represent the medians. The level of statistical significance is indicated as * *p* < 0.05, ** *p* < 0.01, and *** *p* < 0.001.

Rezafungin, anidulafungin, caspofungin, and micafungin in mice infected with isolates of the South Asian, East Asian, South African, and South American clades produced 3–5 log, 1–4 log, 2–4 log, and 2–4 log mean CFU/g decreases, respectively, compared with their respective controls on day 7. The four echinocandins generated at least 3 log mean CFU/g decreases in mice infected with isolates from the South Asian, South African, and South American (bloodstream isolate) clades on day 7 (Figure 2). The kidneys were sterile in three rezafungin-treated mice and two caspofungin-treated mice infected with isolate 12372 (East Asian clade). Anidulafungin generated the lowest (approximately 1 log) CFU/g decrease in mice infected with isolate 12372 (East Asian clade). Moreover, the fungal burden in anidulafungin-treated mice infected with isolate 13112 (environmental isolate from the South American clade) increased by more than 1 log CFU/g compared with controls on day 1. Regardless of the isolates, the average CFU/g in rezafungin- and micafungin-treated mice was lower than that in the controls on day 1 (Figure 2).

#### 3.2.2. Heart

Rezafungin, anidulafungin, caspofungin, and micafungin in mice infected with the five isolates of the four clades produced 2–4 log, 0–4 log, 2–4 log, and 1–4 log mean CFU/g decreases, respectively, compared with their respective controls on day 7. The average fungal burden in anidulafungin-treated mice infected with isolate 13112 (environmental isolate from the South American clade) was the same as that of the control on day 7 (Figure 2). Rezafungin significantly decreased the mean fungal CFU/g burdens in mice infected with all isolates from the four clades. Moreover, for all the isolates in rezafungin-treated mice, the average CFU/g always decreased by at least 1–2 log compared with the controls on day 1 (Figure 2). However, the average fungal burden in the heart was never lower than 10^4^ CFU/g.

#### 3.2.3. Brain

In mice infected with the five isolates, the fungal tissue burden was 1–3 mean log CFU/g higher on day 7 than on day 1 (Figure 2). Although echinocandins, especially caspofungin and micafungin, frequently generated statistically significant CFU/g decreases (1–2 log), the average fungal burden in echinocandin-treated mice ranged from approximately 10^5^ to >10^7^ CFU/g. None of the four echinocandins were effective in mice infected with isolate 13112 (environmental isolate from the South American clade) (Figure 2).

## 4. Histopathology

**Figure 3:**
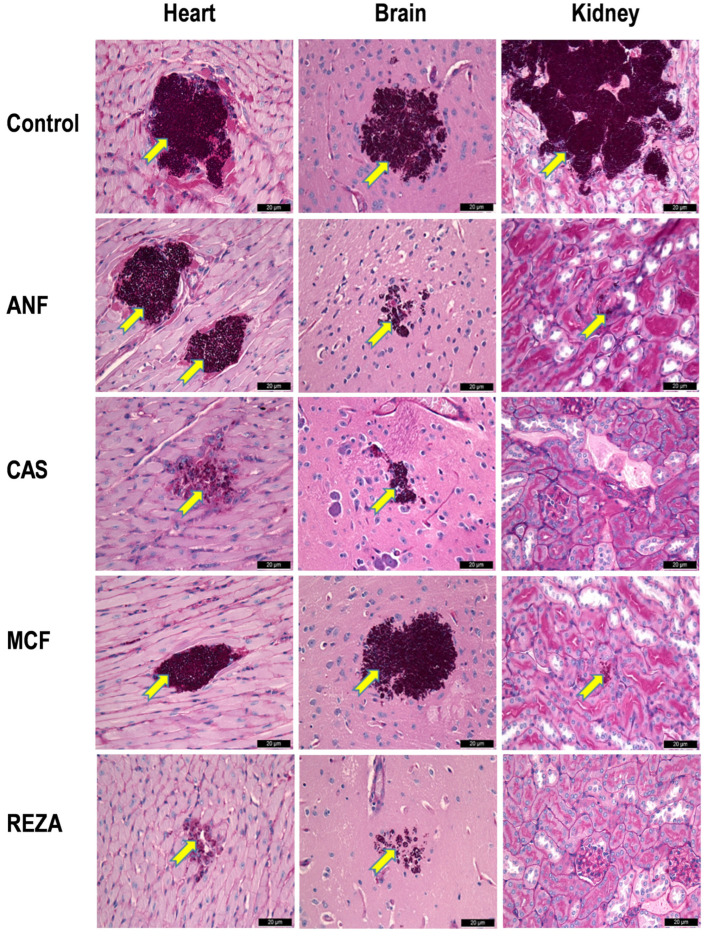
Histopathological findings of the heart, kidney, and cerebrum with periodic acid–Schiff staining in mice infected with isolate CDC B-13112 from the South American clade. A 20 mg/kg dose of rezafungin (REZA) was administered on days 1, 3, and 6. Additionally, beginning 24 h post-infection, the mice received 3 mg/kg of caspofungin (CAS) (Cancidas^®^), 5 mg/kg of micafungin (MCF) (Mycamine^®^), and 5 mg/kg of anidulafungin (ANF) (Eraxis^®^) once daily for 6 days. Fungal lesions are indicated by yellow arrows. In the control mice, *C. auris* produced large aggregates in the heart and kidneys and one large aggregate in the brain. In anidulafungin- and micafungin-treated mice, two and one large aggregates, respectively, with numerous blastoconidia and budding yeast cells were detected in the heart. The size of the lesions in caspofungin-treated mice was similar to that in anidulafungin- and micafungin-treated mice, but the number of fungal cells was significantly lower. The smallest fungal lesions were detected in rezafungin-treated mice. Anidulafungin-, caspofungin-, and rezafungin-treated mice showed small fungal lesions in their brain tissue, but the lesions in micafungin-treated mice were similar to those in the control mice. In the kidneys in caspofungin- and rezafungin-treated mice, fungal cells were not seen, but small lesions were noted in anidulafungin- and micafungin-treated mice. Magnification, ×100.

Histopathologic examination revealed large multifocal fungal infiltrates in the heart, kidneys, and brain of control mice. For isolate 13112, regardless of the echinocandin administered, medium or large foci of fungal cells were detectable in the heart and brain. However, in rezafungin- and caspofungin-treated mice (but not in anidulafungin- and micafungin-treated mice), fungal cells were not detected in the kidneys (Figure 3). For the remaining isolates, despite the moderately high (~10^3^–~10^6^ CFU/g) fungal burden (Figure 2) in all echinocandin-treated mice, fungal cells were not apparent in their kidneys; the exception was mice infected with the clinical (I-156) isolate from the South American clade and treated with micafungin (Supplemental Figure S1). Similarly, for the remaining clinical isolates, mice treated with rezafungin (but not mice treated with the other three echinocandins) showed no fungal cells in their hearts (Supplemental Figure S2). By contrast, regardless of the clades, all echinocandin-treated mice showed medium and/or large foci of fungal cells in their cerebrum or cerebellum (Figure 3 and Figure S3).

## 5. Discussion

In this study, we compared the in vivo efficacies of the four approved echinocandins against 10 clinical isolates belonging to the four main clades of *C. auris* in a neutropaenic murine model. Regardless of the isolates and clades, all four echinocandins significantly prolonged survival. On day 7, the survival rates for the South Asian, East Asian, South African, and South American clades were 80–100%, 40–100%, 60–100%, and 70–100%, respectively, in all treatment arms. In fungal tissue burden experiments, all echinocandins frequently produced >3-log CFU/g decreases in the mean fungal kidney and heart burdens, although some decreases were not statistically significant (Figure 2). However, anidulafungin generated weak CFU/g decreases in the heart and kidneys in mice infected with isolates 12372 (East Asian clade) and 13112 (environmental isolate from the South American clade) (Figure 2). The recently approved rezafungin, regardless of the clades, produced 3–5 and 2–4 log CFU/g decreases in the kidneys and heart, respectively. By contrast, all echinocandins poorly inhibited the fungal growth in the brain, which is consistent with the intrinsically low CNS penetration of this drug class (Figure 2). The histopathological examination findings only in the case of the brain were in line with the results from the lethality and fungal tissue burden experiments (Figure 3 and Figure S3), suggesting that the fungal burden of the kidneys and hearts were not always high enough to be detected by histology.

In previous studies, the micafungin and rezafungin in vivo efficacies against *C. auris* clinical isolates were determined in fungal kidney burden experiments for pharmacodynamic optimization [27,28]. Micafungin at 5 mg/kg daily produced a 2-log kill in neutropaenic mice infected with eight of nine isolates with MICs of ≤2 mg/L [27]. Similar results were obtained in rezafungin-treated neutropaenic mice (20 mg/kg on days 1, 3, and 6 post-infection) infected with three of four *C. auris* isolates with MICs of ≤0.25 mg/L [28]. The same humanized rezafungin and micafungin doses significantly decreased the kidney burden in mice infected with a single *C. auris* isolate by day 7. Moreover, by day 10, rezafungin had produced a significantly lower log CFU/g compared with micafungin [24]. Our current results with rezafungin and micafungin against *FKS* wild-type *C. auris* isolates were similar to these studies. In the previous studies, however, anidulafungin and caspofungin were not tested, the fungal heart and brain burdens were not examined, and neither lethality nor histopathological examinations were performed. The lack of other in vivo studies comparing rezafungin to anidulafungin, caspofungin, and micafungin against the four main *C. auris* clades precludes a comparative discussion of our results.

The main strength of our study is that all four approved echinocandins were tested against wild-type *C. auris* isolates belonging to the four main clades using lethality and fungal tissue burden experiments with a large number of histopathological examinations. Although in our previous in vitro study, the four echinocandins produced moderate to weak fungistatic effects against *C. auris* isolates in RPMI-1640 and 50% serum [15], the echinocandin treatments significantly decreased the fungal burden in the heart and kidneys regardless of the isolate employed. These data suggest that based on MICs, echinocandins would be expected to have activity in vivo against *C. auris* [29,30]. In contrast to previous studies in which echinocandins were administered 2 h post-infection [24,27,28] the echinocandin treatments in the present study were initiated 24 h post-infection, more accurately mimicking clinical situations. However, isolates belonging to the fifth and sixth clades were not tested in this experiment.

A growing body of data suggests that echinocandin exposure (specifically anidulafungin, caspofungin, and micafungin) with the currently used dosing regimens may be low in patients with obesity, critical illness, and peritoneal and pleural space infections, leading to therapeutic failure [4,8,9,10,11,12]. Moreover, Bretagne et al. [31] found that the replacement of fluconazole by echinocandins as the first-line antifungal therapy led to poorer outcomes. Higher echinocandin exposure may increase survival; however, studies have shown that elevated daily doses of caspofungin and micafungin do not significantly increase the cure rates while simultaneously increasing the risk of liver damage [32,33].

Rezafungin is a close structural analogue of anidulafungin and exhibits better stability and excellent tissue penetration. The pharmacokinetic profile of rezafungin in humans is favorable; one study showed that a single dose of 400 mg resulted in a C_max_ of 22.7 ± 3.59 mg/L, AUC_0–168_ of 1160 ± 170 mg·h/L, and half-life of 129 ± 18.6 h, which are significantly higher than those of the three previously approved echinocandins [34]. These beneficial pharmacokinetic properties allow for the once-weekly administration of rezafungin, and the high front-loaded plasma exposure offers earlier concentration-dependent killing and mycological clearance as shown in prospective randomized controlled trials [35]. However, clinical data regarding the efficacy of rezafungin in patients with candidaemia or invasive candidiasis caused by *C. auris* are lacking.

In this study, consistent with previous studies, the recently approved rezafungin showed comparable to or better in vivo activity than anidulafungin, caspofungin, and micafungin against *C. auris* isolates. In the heart, regardless of the isolates, only rezafungin-treated mice showed a 1–2 log decrease in the fungal burden compared with day 1. Although the heart is one of the organs in which the echinocandin tissue/plasma AUC ratio is low (approximately 1) [36], the high front-loaded plasma exposure of rezafungin was high enough to significantly decrease the heart burden. These findings are notable because heart and kidney failure is predictive of imminent death in mice with disseminated candidiasis [37].

Despite the negligible in vitro killing activity of caspofungin against *C. auris* clades in our previous study, the caspofungin activity in the lethality and fungal tissue burden experiments was comparable to that of rezafungin [15]. A possible explanation for the efficacy of caspofungin against *C. auris* isolates may be related to its relatively lower protein binding (96.5%) compared to other echinocandins (i.e., ≥99% for anidulafungin or micafungin) [6]. Moreover, no rezafungin- or caspofungin-treated mice showed large fungal cell aggregates in the hearts and kidneys, suggesting better eradicating activity of these two antifungals compared with anidulafungin and micafungin. These data confirm that a direct correlation does not necessarily exist between in vitro and in vivo studies. The current results are in line with phase II and III clinical studies in which rezafungin was non-inferior to caspofungin in the treatment of candidaemia and invasive candidiasis caused by *C. albicans*, *C. glabrata*, *C. tropicalis*, and *C. parapsilosis* [35].

Candidaemia due to *C. auris* is a life-threatening infection that requires prompt treatment with echinocandins. Treatment failure with echinocandins, especially in patients with COVID-19, is associated with mortality rates of 67% to 83% [38,39]. Moreover, persistent candidaemia due to *C. auris* was observed in four of six patients who died despite having received a standard dose of micafungin therapy for 5 days, suggesting an inadequate clearance of the fungus from the bloodstream [39]. The initiation of early treatment with the next-generation echinocandin rezafungin provides high plasma drug exposure early in the course of treatment with a higher killing rate, decreasing the risk of persistent candidaemia and metastatic complications (i.e., endophthalmitis, spondylodiscitis, and endocarditis/myocarditis) as observed in phase II and III clinical studies [35]. Based on a population pharmacokinetic model for *C. auris*, the simulated probability of target attainment (*f*AUC_0–168h_/MIC ratio) for stasis and a 1-log CFU/g decrease following a 400 mg dose of rezafungin was >90% at MIC values of ≤0.25 mg/L. This suggests that the currently used dosing regimen of rezafungin will produce adequate exposure in patients with invasive *C. auris* infection [13,40]. Our current results support rezafungin as a therapeutic option for the treatment of *C. auris*-induced candidaemia or invasive candidiasis with kidney and heart involvement. By contrast, echinocandins poorly inhibited fungal growth in the brain (Figure 2), reinforcing the current recommendations against echinocandins for the treatment of fungal meningoencephalitis [4,13].

## 6. Conclusions

In summary, consistent with prior echinocandin in vitro data demonstrating activity against WT strains, this class was highly efficacious in vivo against *C. auris*. Based on the lethality experiments, fungal kidney and heart burden experiments, and histopathological examinations, rezafungin activity was comparable to or better than that of the three previously approved echinocandins, regardless of clade. Outcome data from the use of rezafungin to treat patients with *C. auris* infections will be informative to determine if these in vivo efficacy trends translate clinically.

## Figures and Tables

**Table 1:** MIC values of rezafungin (REZA), anidulafungin (ANF), caspofungin (CAS), and micafungin (MCF) in RPMI-1640 against *Candida auris* isolates. MICs were determined three times using the CLSI broth microdilution method.

Isolates Number	Clade	Body Site	Aggregation	MIC Values (mg/L)			
				REZA	ANF	CAS	MCF
27 (NCPF 8991)	South Asian	Pleural fluid	−	0.12	0.12	0.5	0.25
196	South Asian	Blood	−	0.25	0.12	0.5	0.25
12372 (CBS 12372)	East Asian	Blood	+	0.06	0.03	0.12	0.03
12373 (CBS 12373)	East Asian	Blood	+	0.12	0.06	0.25	0.06
2 (NCPF 8977)	South African	Cerebrospinal fluid	+	0.12	0.03	0.5	0.25
204	South African	Tracheostomy	+	0.06	0.03	0.25	0.12–0.25
I-24	South American (Israel)	Blood	−	0.25	0.06	0.25	0.12
I-156	South American (Israel)	Blood	−	0.25	0.06	0.5	0.12
13108 (CDC B-13108)	South American (Colombia)	Hospital environment	−	0.12	0.06	0.25	0.12
13112 (CDC B-13112)	South American (Colombia)	Hospital environment	−	0.25	0.12	0.25	0.12

## Data Availability

The original contributions presented in the study are included in the article/supplementary material, further inquiries can be directed to the corresponding author.

## Supplementary Materials

The following supporting information can be downloaded at: https://www.mdpi.com/article/10.3390/jof10090617/s1. Figure S1: Histopathological examination of the kidneys using periodic acid–Schiff staining in neutropaenic mice intravenously challenged with *C. auris* isolates 196 (South Asian clade), CBS 12372 {12372} (East Asian clade), 2 (South African clade), and I-156 (South American clade). A 20 mg/kg dose of rezafungin (REZA) was administered on days 1, 3, and 6. Additionally, beginning 24 h post-infection, the mice received 3 mg/kg of caspofungin (CAS) (Cancidas^®^), 5 mg/kg of micafungin (MCF) (Mycamine^®^), and 5 mg/kg of anidulafungin (ANF) (Eraxis^®^) once daily for 6 days. Histopathological examination was performed 7 days post-infection. Fungal lesions are indicated by yellow arrows. Control mice exhibited both larger aggregates (isolates 196 and I-156) and smaller aggregates (isolates 12372 and 2). In echinocandin-treated mice, with the exception of mice infected with isolate I-156 and treated with micafungin, fungal cells were not seen in the kidneys. Magnification, ×100. Figure S2: Histopathological examination of hearts using periodic acid–Schiff staining from neutropaenic mice intravenously challenged with *C. auris* isolates 196 (South Asian clade), CBS 12372 {12372} (East Asian clade), 2 (South African clade), and I-156 (South American clade). A 20 mg/kg dose of rezafungin (REZA) was administered on days 1, 3, and 6. Additionally, beginning 24 h post-infection, the mice received 3 mg/kg of caspofungin (CAS) (Cancidas^®^), 5 mg/kg of micafungin (MCF) (Mycamine^®^), and 5 mg/kg of anidulafungin (ANF) (Eraxis^®^) once daily for 6 days. Histopathological examination was performed 7 days post-infection. Fungal involvement is indicated by yellow arrows. Coagulative necrosis of myocytes indicates previous infiltration of fungal cells. Large fungal lesions (yellow arrows) were detectable in anidulafungin-treated mice (isolate 12372 from the East Asian clade) and micafungin-treated mice (isolate 196 from the South Asian clade). Regardless of isolates, no fungal cells were detectable in rezafungin-treated mice. In mice treated with the remaining echinocandins, only small fungal lesions were detected (yellow arrows) or fungal cells were not seen. Magnification, ×100. Figure S3: Histopathological examination of brains using periodic acid–Schiff staining from neutropaenic mice intravenously challenged with *C. auris* isolates 196 (South Asian clade), CBS 12372 {12372} (East Asian clade), 2 (South African clade), and I-156 (South American clade). A 20 mg/kg dose of rezafungin (REZA) was administered on days 1, 3, and 6. Additionally, beginning 24 h post-infection, the mice received 3 mg/kg of caspofungin (CAS) (Cancidas^®^), 5 mg/kg of micafungin (MCF) (Mycamine^®^), and 5 mg/kg of anidulafungin (ANF) (Eraxis^®^) once daily for 6 days. Histopathological examination was performed 7 days post-infection. Fungal involvement is indicated by yellow arrows. Small, medium, or large fungal lesions (yellow arrows) were detectable in all treated mice. The largest lesions were found in caspofungin-treated mice (isolate 196 from the South Asian clade). Magnification, ×400.
