# Supplementary figures and images for: In Vivo Efficacy of Rezafungin, Anidulafungin, Caspofungin, and Micafungin against Four Candida auris Clades in a Neutropenic Mouse Bloodstream Infection Model
Source: J Fungi (Basel). 2024 Aug 29;10(9):617. doi: 10.3390/jof10090617 (PMC11433204; doi:10.3390/jof10090617)

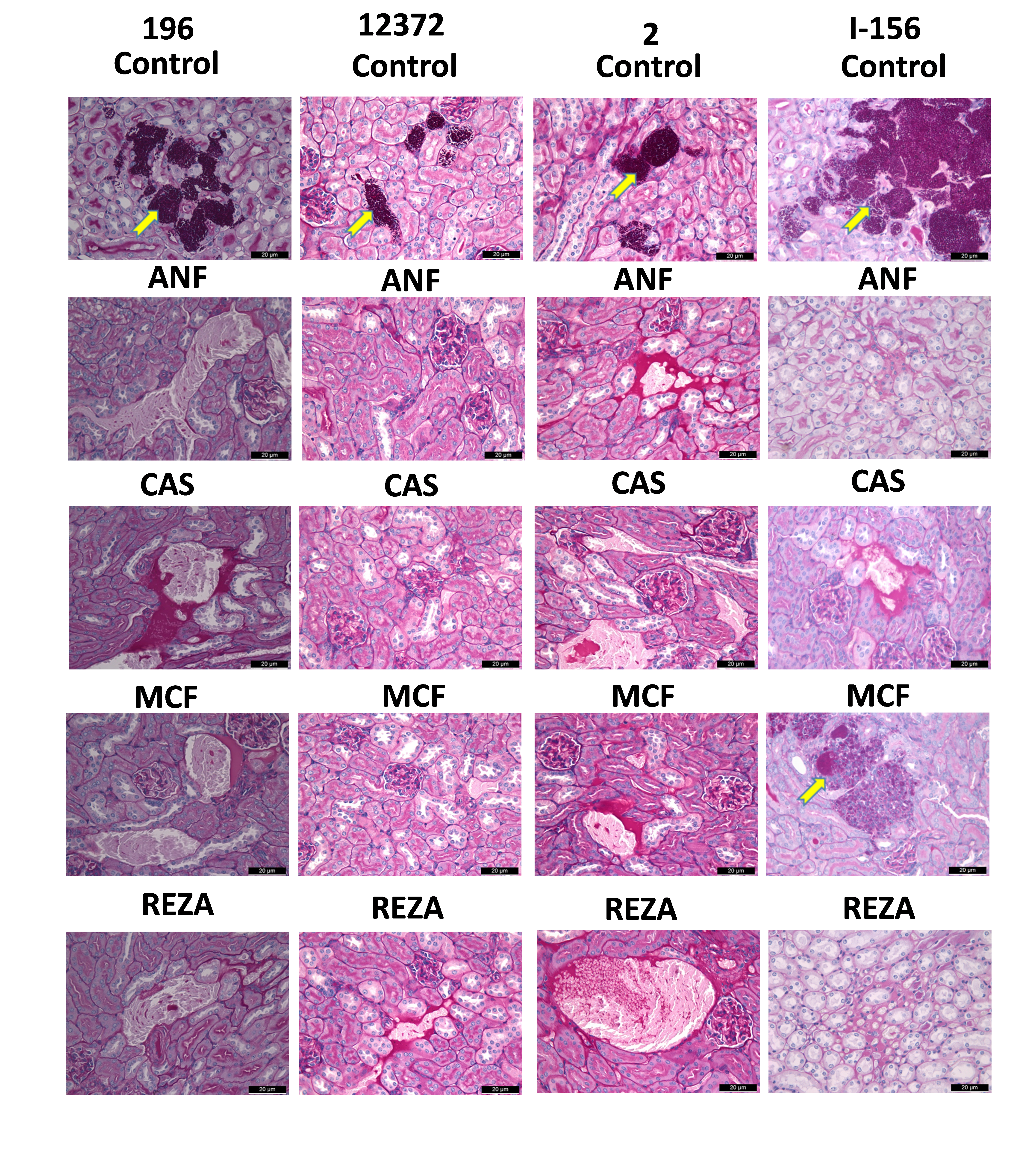

Supplement: Supplementary file 1 [file jof-10-00617-s001.zip › Figure S1.tif]

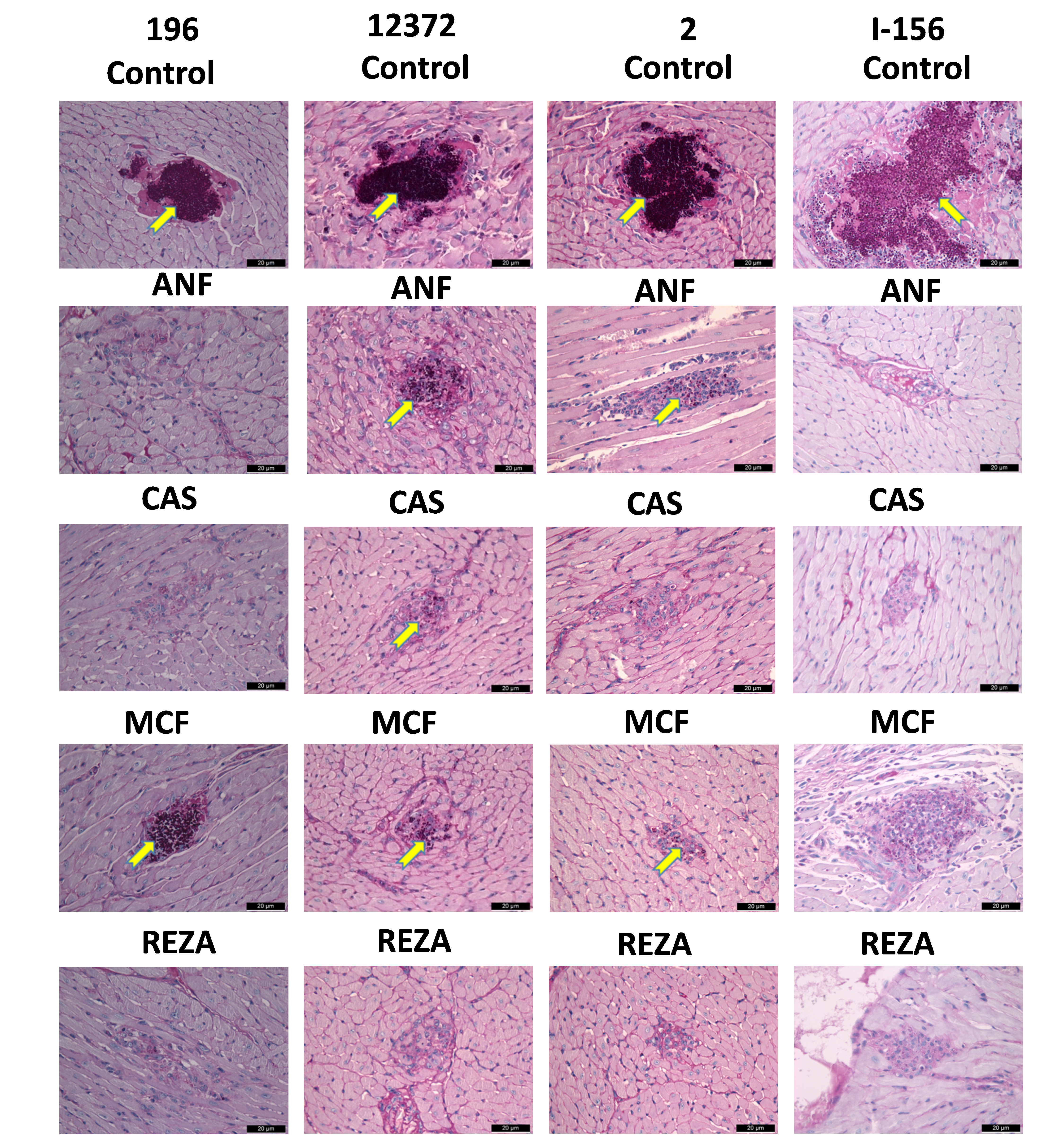

Supplement: Supplementary file 1 [file jof-10-00617-s001.zip › Figure S2.tif]

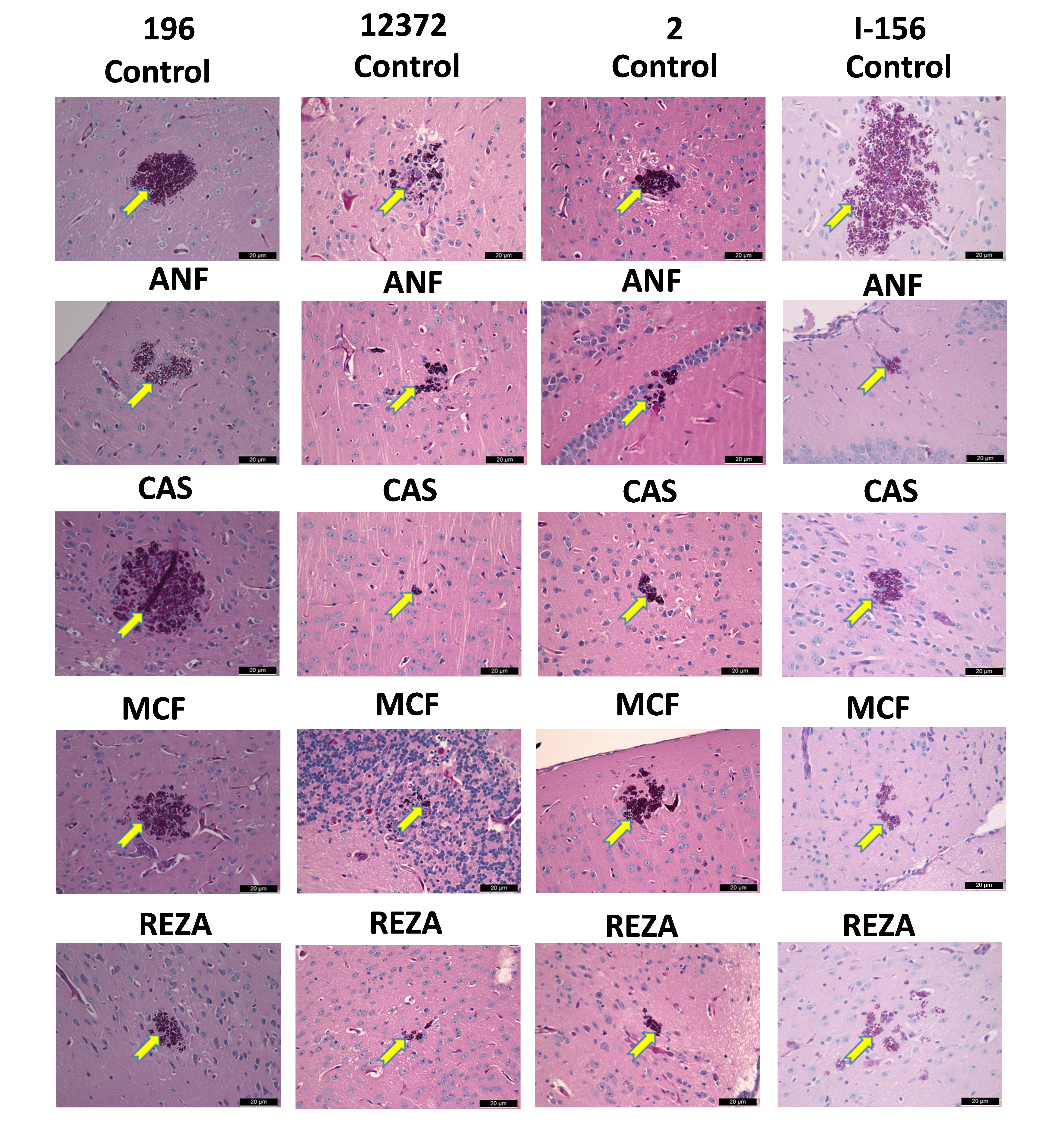

Supplement: Supplementary file 1 [file jof-10-00617-s001.zip › Figure S3.tif]
